# Secure Communications in CIoT Networks with a Wireless Energy Harvesting Untrusted Relay

**DOI:** 10.3390/s17092023

**Published:** 2017-09-04

**Authors:** Hequn Hu, Zhenzhen Gao, Xuewen Liao, Victor C. M. Leung

**Affiliations:** 1 School of Electronic and Information Engineering, Xi’an Jiaotong University, No. 28 West Xianning Road, Xi’an 710049, China; huhequn123@stu.xjtu.edu.cn (H.H.); yeplos@xjtu.edu.cn (X.L.); 2 Department of Electrical and Computer Engineering, The University of British Columbia, Vancouver, BC V6T1Z4, Canada; vleung@ece.ubc.ca

**Keywords:** physical layer security, CIoT networks, untrusted relay, destination-aided jamming, wireless energy harvesting

## Abstract

The Internet of Things (IoT) represents a bright prospect that a variety of common appliances can connect to one another, as well as with the rest of the Internet, to vastly improve our lives. Unique communication and security challenges have been brought out by the limited hardware, low-complexity, and severe energy constraints of IoT devices. In addition, a severe spectrum scarcity problem has also been stimulated by the use of a large number of IoT devices. In this paper, cognitive IoT (CIoT) is considered where an IoT network works as the secondary system using underlay spectrum sharing. A wireless energy harvesting (EH) node is used as a relay to improve the coverage of an IoT device. However, the relay could be a potential eavesdropper to intercept the IoT device’s messages. This paper considers the problem of secure communication between the IoT device (e.g., sensor) and a destination (e.g., controller) via the wireless EH untrusted relay. Since the destination can be equipped with adequate energy supply, secure schemes based on destination-aided jamming are proposed based on power splitting (PS) and time splitting (TS) policies, called intuitive secure schemes based on PS (Int-PS), precoded secure scheme based on PS (Pre-PS), intuitive secure scheme based on TS (Int-TS) and precoded secure scheme based on TS (Pre-TS), respectively. The secure performances of the proposed schemes are evaluated through the metric of probability of successfully secure transmission ($P_{SST}$), which represents the probability that the interference constraint of the primary user is satisfied and the secrecy rate is positive. $P_{SST}$ is analyzed for the proposed secure schemes, and the closed form expressions of $P_{SST}$ for Pre-PS and Pre-TS are derived and validated through simulation results. Numerical results show that the precoded secure schemes have better $P_{SST}$ than the intuitive secure schemes under similar power consumption. When the secure schemes based on PS and TS polices have similar $P_{SST}$, the average transmit power consumption of the secure scheme based on TS is lower. The influences of power splitting and time slitting ratios are also discussed through simulations.

## 1. Introduction

Internet of Things (IoT) represents an emerging era of networking that provides ubiquitous connectivity and information exchange spanning home, vehicular, healthcare monitoring and industrial environment [1,2,3,4]. A large number of common entities with computing and communication capabilities can be connected to the Internet. The pervasive sensing and control capabilities of such smart objects will lead to a transformative change of the whole society. Although the term IoT has been proposed for almost a decade [5], the corresponding technologies and protocols are still open research issues.

A serious issue caused by the usage of massive IoT devices is the spectrum scarcity. The concept of Cognitive Internet of Things (CIoT) has been advocated to solve this problem [6,7,8,9,10,11]. In a CIoT network, an IoT device acts as an unlicensed secondary user and operates on the same spectrum bands owned by a licensed primary user. According to the sensing ability of the IoT device, the IoT device can access the spectrum in different ways. When the IoT device has the capability of spectrum sensing, it senses the spectrum and transmits when a spectrum whole is detected. This kind of dynamic spectrum access is called overlay spectrum sharing [12]. However, it is challenging to design lightweight spectrum sensing algorithms with high detection probability for a simple and energy-constraint device. When the IoT device does not sense the spectrum, it accesses the primary spectrum bands as long as the secondary transmission satisfies the interference threshold constraint [12], which indicates the tolerance of the secondary transmission at the primary receiver [13]. This kind of dynamic spectrum access is called underlay spectrum sharing [10]. To satisfy the interference threshold constraint, it is required that the secondary user has the knowledge of the interference level at the primary receiver. Spectrum leasing is another way to access the primary spectrum bands when the IoT device does not employ spectrum sensing [9,11]. In CIoT networks based on spectrum-leasing, the primary user leases its spectrum to the secondary user who has helped to relay the primary signals. Despite the limited resources including restricted power supply, limited data processing capability and range of communication, the IoT devices have to share their precious resources with the primary user to win the rights to access the primary spectrum.

Communication security is obviously another critical problem in IoT networks, due to their extensive application in commercial, governmental, industrial and military applications [14]. However, the broadcast nature of wireless communication makes the transmission vulnerable to eavesdropping attack. Traditional cryptographic encryption has been widely used to protect the message from being eavesdropped [15,16]. Nevertheless, there are difficulties and vulnerabilities associated with key distribution and management in IoT networks that have a very large number of resource-constrained IoT devices, heterogeneous Radio access technologies (RATs) and different subsystems controlled by distinct operators. As a result, lightweight protocols with high efficiency are appealing solutions for the security issues in the IoT.

Physical layer security (PLS) has attracted much attention recently, since it is generally irrelevant to the RAT, and offers “built-in” security that is information-theoretically unbreakable. The main idea of PLS is exploiting the wireless channels and interference environments to keep the confidential message from eavesdropping. So far, a variety of PLS techniques have been proposed, such as artificial noise techniques [17], cooperative relay transmission [18], secure beamforming [19], and coding strategy [20]. Since cooperative communication through the relays has proven advantageous in improving the network coverage and energy efficiency, PLS techniques based on cooperative relay transmission are of significant importance in the IoT, where the devices usually have restricted power supply and limited coverage range for the reliable communication. Numerous papers have emerged to deal with the secrecy issue in relay networks where the relays are trusted and act as friendly helpers to resist external eavesdroppers [18,21,22]. However, the relay itself should be considered as an untrusted entity in some applications. For example, in defense, financial, and government intelligence networks, different users have the different rights to access information. Furthermore, a relay from a different network may not have the permission to acquire the information as the source and the destination does. How to keep the information confidential from the relay is an important security issue.

Taking into consideration the severe issues of spectrum scarcity and security, a CIoT network is considered and underlay spectrum sharing strategy is used, where sensing capability is not required for the IoT devices. A relay acts as an information forwarder as well as a potential eavesdropper. Without considering the energy constraint of the relay, numerous papers have studied the secure transmission via the relays [23,24]. Since energy harvesting techniques can exploit the external energy source and relieve devices from the constraints induced by battery usage, a prospective study on the secure transmission via an energy harvesting relay is provided in this paper. To enhance the security of the CIoT network, the destination transmits jamming signals to jam the untrusted relay while the IoT device transmits the information signals. Both the information and jamming signals are used by the EH relay for energy harvesting. Power splitting (PS) and time splitting (TS) receiver architectures [25] are used at the relay. Our main contributions and key results are summarized in the following.

- We propose PLS transmission schemes for a CIoT network where an untrusted relay helps the secondary transmission. To the best of our knowledge, this is the first paper to consider the security issue in an underlay CIoT network with an untrusted relay from the PLS perspective. Since the transmissions of the IoT nodes may cause interference with the primary receiver, the existing secrecy criteria (e.g., secrecy outage or secrecy rate) can not describe the system performance properly. In this paper, we derived a new criterion to illustrate the secrecy performance of the CIoT network.
- To protect the information from being intercepted by the untrusted relay, amplify-and-forward relaying protocol is used and destination-aided jamming strategy is adopted. An intuitive secure scheme and a precoded secure scheme are proposed for the CIoT network based on PS and TS policies, respectively. The secrecy performances of these schemes are evaluated by the probability of successfully secure transmission ($P_{SST}$), which represents the probability that the interference threshold constraint is satisfied and the secrecy rate of the secondary transmission is positive. The closed forms of $P_{SST}$ of the precoded secure schemes based on PS and TS policies are given and verified with the simulation results.
- We compare the intuitive secure scheme and the precoded secure scheme based on PS and TS policies, and find out that the precoded secure schemes have better $P_{SST}$ than the intuitive secure schemes under similar power consumption. Moreover, the precoded secure scheme based on TS policy is more energy efficient than that based on PS policy.
- The numerical results show that $P_{SST}$ of the PS policy is not sensitive to the PS ratio when $P_{SST}$ reaches a certain value, and an optimal PS ratio maximizing the achievable secrecy rate is considered under the $P_{SST}$ constraint. In the TS policy, the time splitting ratio shows both constructive and destructive effects on the two-hop secondary transmission via the EH untrusted relay. Thus, there exists an optimal energy harvesting time in the TS policy that maximizes $P_{SST}$.

The rest of the paper is organized as follows. We discuss the related work in Section 2. Section 3 describes the CIoT network with an untrusted EH relay. The PLS schemes based on PS and TS policies are proposed in Section 4 and Section 5, respectively, where the performances of the proposed PLS schemes are analyzed in terms of $P_{SST}$. Numerical results are presented in Section 6, and the effects of different system parameters on the secrecy performance of the proposed PLS schemes are discussed and various design insights are obtained. Finally, we draw conclusions in Section 7.

## 2. Related Work

The related research about physical layer security suitable for IoT is summarized in this section. Then, we discuss some existing work on PLS using untrusted relays and energy harvesting entities.

There are two main categories of PLS techniques: (1) intelligent designs to keep the information secure from the eavesdroppers where no secret key is needed; and (2) generation of secret keys over public channels by exploiting the wireless communication medium [26]. In this paper, we are more interested in the first category, which does not require error-free two-way public channels. Moreover, keyless secrecy methods are more easily extended to large-scale sensor networks.

In the downlink communication network of the IoT, the controllers transmit signals, and they could be equipped with multiple antennas and adequate energy supply. The PLS schemes such as optimal precoding, artificial noise and secure space-time coding can be applicable in the IoT, and the pros and cons of these conventional PLS techniques have been summarized in [14]. Secrecy rate and secrecy outage probability are main metrics to evaluate the secrecy performance. In the uplink communication network of the IoT, an IoT device, such as a sensor or a surveillance camera, transmits to the controller, and the IoT device is usually resource-constrained. The channel-aware encryption (CAE) scheme proposed in [27] is an appealing solution in sensor networks where sensors have very low data rate. In the CAE scheme, a sensor may stay dormant, report a “flipped” decision, or report its unaltered local decision at each instant. How it acts depends on where its instantaneous channel fading gain to the legal controller falls among some known thresholds. How to optimize these comparison thresholds is not discussed by the authors in [27]. In [28], the optimal thresholds were derived to further improve the performance. When relays are used in IoT networks with passive eavesdroppers with locations, a randomize-and-forward relay scheme has been proposed in [29]. The authors formulated a secrecy-rate maximization problem subject to a secrecy-outage-probability constraint, and designed the optimal power allocation and codeword rate [29]. Considering the spectrum scarcity, a Cognitive Internet of Things (CIoT) has been proposed where the IoT device acts as a secondary user and accesses the primary spectrum by using the spectrum-leasing strategy [9]. To achieve secure transmission, the authors utilized cooperative jamming performed by an energy harvesting helper. Based on the cooperative jamming scheme, an auction framework was proposed to build an incentive mechanism for the secondary users. The channel assignment problem in time-critical IoT-based cognitive radio networks under proactive jamming attacks was considered in [30]. Subject to delay constraints, a probabilistic spectrum assignment algorithm that aimed at minimizing the packet invalidity ratio of each cognitive radio transmission has been proposed. Since energy harvesting is an appealing and promising technology [31,32], more and more papers study PLS problems with EH nodes recently. In [10], the PLS issue of cognitive sensor radio networks (CSRNs) with an external EH eavesdropper was investigated. Underlay spectrum sharing was used in CSRNs. The sensor node acts as the secondary user, and adjusts its transmit power to guarantee the primary user’s quality-of-service (QoS). Two scenarios with different interference power constraints were studied and the closed-form analytical expressions of secrecy outage probability for both cases were derived [10]. Authors in [33] considered an underlay cognitive radio system, where a source in a secondary system transmitted information to a full-duplex (FD) wireless EH destination node in the presence of an eavesdropper. The harvested energy at the destination was used to send jamming signals, so that the eavesdropper’s decoding capacity is degraded. Upper and lower bounds of probability of strictly positive secrecy capacity (SPSC) have been derived in [33]. However, these existing secure schemes are based on the assumption that the nodes in the IoT or CIoT are trusted, and they are designed to prevent interception from the eavesdroppers outside.

When untrusted relays are considered, numerous PLS schemes have been proposed based on different relaying protocols. Authors in [34] adopted a successive amplify-and-forward (AF) relaying scheme, where the multi-antenna source transmitted to two selected nodes alternately. The inter-relay interference, which is usually regarded as detrimental, was used to jam the untrusted nodes. The authors proposed several relay selection schemes with different complexities and derived the closed-form expressions of the lower bound of secrecy outage probability in [34]. For multiple-antenna untrusted relay systems, a joint destination-aided cooperative jamming and precoding scheme was devised to maximize the secrecy rate by jointly designing the precoding matrices for the source, relay, and destination [35]. Authors in [23] proposed a modulo-and-forward (MF) protocol at the relay with nested lattice encoding at the source to improve the secrecy in a dual-hop untrusted relay network. A multi-hop line network was considered in [24], where each node received signals transmitted by its neighbors, and the leftmost node sent messages to the rightmost node. When any or all of the relay nodes can be eavesdroppers, it has been shown in [24] that it is possible to achieve end-to-end secure and reliable communication by utilizing nested lattice codes. Kalamkar, S.S. et. al. in [32] investigated the problem of secure cooperative communication with the help of a wireless EH untrusted node. To realize the positive secrecy rate, destination-aided cooperative jamming was used. Analytical expressions were derived for the secrecy outage probability and the ergodic secrecy rate to evaluate the secrecy performance in [32].

The goal of this paper is to solve the security problem in a CIoT network where a wireless EH untrusted node is used to relay the IoT device’s information. Underlay spectrum sharing is adopted by the CIoT network to relieve the stress of spectrum scarcity. As long as the interference threshold constraint is satisfied, the IoT device and the relay can access the primary spectrum, and the capability of spectrum sensing is not required. Although the IoT device and the relay may have strict energy constraints, the controller could have adequate energy supply. Therefore, destination-aided cooperative jamming is used to provide secure transmission. Together with the source signal, this jamming signal is also used for energy harvesting at the relay.

## 3. System Model

**Figure 1:**
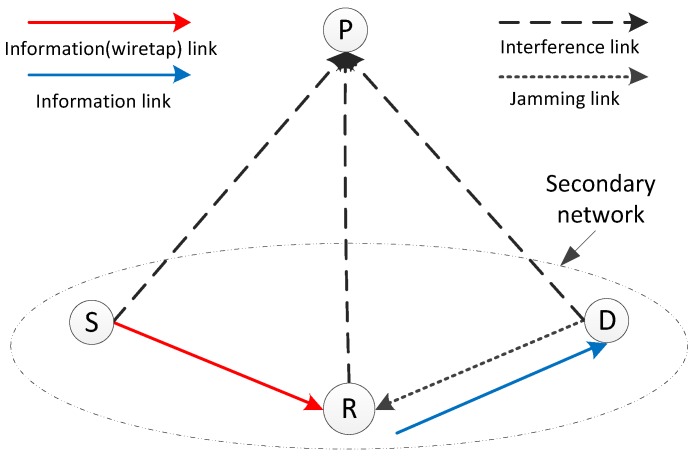
A brief system model of the CIoT network.

The considered CIoT network is shown in Figure 1, where an IoT network works as the secondary network, and the primary network has a primary receiver *P*. The primary transmitter is located far away from the IoT network as in [10,33]. Therefore, there is no interference from the primary transmitter to the IoT network. In the secondary IoT network, an IoT device *S* (e.g., sensor) tries to transmit to the destination *D* (e.g., controller) through an EH untrusted relay node *R*. The direct link between *S* and *D* is unavailable. Although the untrusted relay helps the secondary transmission, *S* and *D* try to prevent information leakage to the relay. Each node is equipped with a single antenna and works in half-duplex mode. Channel reciprocity is assumed as in [32]. It is assumed that all links experience independent and quasi-static Rayleigh fading, and the channel remains constant during the period of *T* [32]. The channel power gain is given by $∥h_{c}{∥}^{2}$, which has exponential distribution with mean $g_{c}$ , i.e.,

(1) $$f_{|h_{c}{|}^{2}}\left(x\right)=\frac{1}{g_{c}}e^{-\frac{x}{g_{c}}},$$

where $h_{c}$ represents the link between S−R, D−R, S−P, D−P or R−P, the subscript *c* can be SR, DR, SP, DP, and RP accordingly, and $f_{|h_{c}{|}^{2}}\left(x\right)$ is the probability density function of random variable $|h_{c}{|}^{2}$.

**Figure 2:**
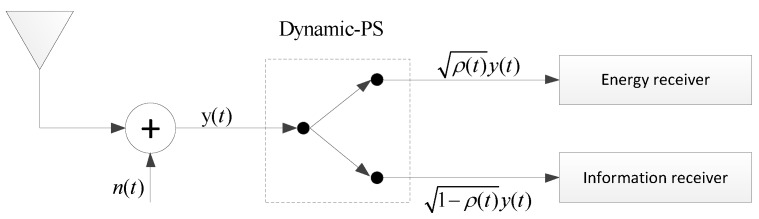
Architecture for the separated information and energy receiver [25].

For secondary transmissions, the interference with the primary receiver *P* is required to be under the interference threshold Γ. Assume that the secondary nodes know the statistic channel information between them and the primary receiver [36]. As in [31,37], the relay uses the harvested energy completely for the transmission. PS and TS based receiver-architecture are used at *R*, and the receiver architecture for the separated information and energy receiving is shown in Figure 2. With PS, the information receiver and the energy receiver are both in on mode for a duration of *T*. The relay splits the received power for two purposes: one part for energy harvesting and the remaining part for information processing. With TS, the relay splits the time of *T*, and switches between the status of energy harvesting and information processing.

It should be pointed out that *R* is untrusted, and it may attempt to decode the source information while relaying the information. In the following, we will give secure relaying schemes based on PS and TS policies. Unless otherwise stated, the notations are consistent in this paper.

## 4. Secure Schemes Based on PS Policy

**Figure 3:**
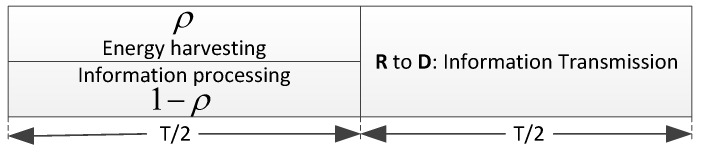
The PS policy.

As shown in Figure 3, a transmission period of *T* for the PS policy is divided into two phases with equal durations. In the first phase, *S* and *D* transmit simultaneously to *R* with power $P_{S}$ and $P_{D}$ respectively. The jamming signal transmitted by *D* is used not only as an interference but also as an energy source to *R*. *R* uses ρ (0<ρ<1) of the received power for energy harvesting and the rest (1−ρ) of the received power for information processing. ρ is the power splitting ratio. By exploiting the harvested energy, *R* amplifies and forwards the received information to *D* in the second phase. An intuitive secure scheme based on PS policy (Int-PS) is given as follows.

### 4.1. Intuitive Secure Scheme Based on PS Policy

#### 4.1.1. Energy Harvesting and Information Processing of Int-PS

The harvested energy $E_{H}$ at the relay from the first phase of the PS policy can be written as

(2) $$E_{H}=\eta \rho (P_{S}|h_{SR}{|}^{2}+P_{D}|h_{DR}{|}^{2})\left(\frac{T}{2}\right),$$

where η∈(0,1] is the energy transform efficiency, whose value depends on the energy harvesting circuit design of *R*. The terms $P_{S}{\left|h_{SR}\right|}^{2}$ and $P_{D}{\left|h_{DR}\right|}^{2}$ in (2) represent the power received at *R* from *S* and *D*, respectively. Then, *R* uses the harvested energy to forward the source information to *D* in the second phase, and the transmit power of *R* becomes

(3) $$P_{H}=\frac{E_{H}}{T/2}=\eta \rho (P_{S}|h_{SR}{|}^{2}+P_{D}|h_{DR}{|}^{2}).$$

*R* uses the remaining (1−ρ) of the received signal in the first phase for information processing. Denote this part of signal as $y_{R}$, and $y_{R}$ is expressed as

(4) $$y_{R}=\sqrt{\left(1-\rho \right)P_{S}}h_{SR}x_{S}+\sqrt{\left(1-\rho \right)P_{D}}h_{DR}x_{D}+n_{R},$$

where $x_{S}$ is the information signal of unit power, $x_{D}$ is the jamming signal with unit power transmitted from *D*, $n_{R}$ is the additive white Gaussian noise (AWGN) at *R*, and $n_{R}∼\mathrm{CN}\left(0,\sigma_{R}^{2}\right)$. The untrusted relay may try to intercept the information message $x_{S}$ of *S*. Based on (4), the signal to interference and noise ratio (SINR) at *R* can be given as

(5) $$\gamma_{R}^{Int-PS}=\frac{\left(1-\rho \right)P_{S}{\left|h_{SR}\right|}^{2}}{\left(1-\rho \right)P_{D}{\left|h_{DR}\right|}^{2}+\sigma_{R}^{2}}.$$

Similarly, the received signal $y_{p1}$ at the primary user *P* is

(6) $$y_{P1}=\sqrt{P_{S}}h_{SP}x_{S}+\sqrt{P_{D}}h_{DP}x_{D}+n_{P1},$$

where $n_{P1}$ is the AWGN at *P* in the first phase, $n_{P1}∼\mathrm{CN}\left(0,\sigma_{P1}^{2}\right)$. The received power of interference plus AWGN at *P* is given as

(7) $$P_{I1}^{Int-PS}=P_{S}|h_{SP}{|}^{2}+P_{D}{\left|h_{DP}\right|}^{2}+\sigma_{P1}^{2}.$$

In the second phase, *R* amplifies $y_{R}$ as $x_{R}=\beta y_{R}$, and forwards $x_{R}$ to *D*, where $\beta =\sqrt{\frac{P_{H}}{\left(1-\rho \right)(P_{S}|h_{SR}{|}^{2}+P_{D}|h_{DR}{|}^{2})+\sigma_{R}^{2}}}$. The received signal $y_{D}$ at *D* is

(8) $$\begin{array}{cc} y_{D} & =h_{RD}x_{R}+n_{D}\\ & =h_{RD}\beta \sqrt{\left(1-\rho \right)}\left(\sqrt{P_{S}}h_{SR}x_{S}+\sqrt{P_{D}}h_{DR}x_{D}\right)+h_{RD}\beta n_{R}+n_{D}, \end{array}$$

where $n_{D}$ is the AWGN at *D*, $n_{D}∼\mathrm{CN}\left(0,\sigma_{D}^{2}\right)$. The interference term $h_{RD}\beta \sqrt{\left(1-\rho \right)P_{D}}h_{DR}x_{D}$ in (8) can be cancelled by *D* since the jamming signal $x_{D}$ was sent by *D* itself. After the self-interference cancellation, the remainging signal $y_{D}^{\prime }$ at *D* becomes

(9) $$y_{D}^{\prime }=\beta \sqrt{\left(1-\rho \right)P_{S}}h_{SR}h_{DR}x_{S}+\beta h_{DR}n_{R}+n_{D}.$$

The signal to noise (SNR) at *D* can be written as

(10) $$\gamma_{D}^{Int-PS}=\frac{\beta^{2}\left(1-\rho \right)P_{S}|h_{SR}{|}^{2}{\left|h_{DR}\right|}^{2}}{\beta^{2}{\left|h_{DR}\right|}^{2}\sigma_{R}^{2}+\sigma_{D}^{2}}.$$

Substituting β in (10), the SNR at *D* is rewritten as

(11) $$\gamma_{D}^{Int-PS}=\frac{\eta \rho \left(1-\rho \right)P_{S}|h_{SR}{|}^{2}{\left|h_{DR}\right|}^{2}}{\eta \rho |h_{DR}{|}^{2}\sigma_{R}^{2}+\left(1-\rho \right)\sigma_{D}^{2}+\frac{\sigma_{R}^{2}\sigma_{D}^{2}}{P_{S}|h_{SR}{|}^{2}+P_{D}{\left|h_{DR}\right|}^{2}}}.$$

Finally, we can get the instantaneous power interference to *P* in the second phase as

(12) $$P_{I2}^{Int-PS}=\eta \rho (P_{S}|h_{SR}{|}^{2}+P_{D}|h_{DR}{|}^{2})|h_{RP}{|}^{2}+\sigma_{P2}^{2},$$

where $\sigma_{P2}^{2}$ is the power of the noise at *P* in the second phase.

#### 4.1.2. Probability of Successfully Secure Transmission of Int-PS

Since *R* is untrusted, the instantaneous secrecy rate $R_{S}$ of the secondary IoT network can be written as [38]

(13) $$R_{S}^{PS}=\frac{1}{2}{\left[\log_{2}\left(\frac{1+\gamma_{D}}{(1+\gamma_{R}}\right)\right]}^{+},$$

where ${\left[x\right]}^{+}=max\left(x,0\right)$. In the CIoT network, $P_{SST}$ represents the probability of successfully secure transmission. Note that “the successfuly secure transmission” represents that only when the total interference power at the primary user is under the interference threshold can the source transmit its message to the destination, while the secrecy rate is greater than zero. Therefore, $P_{SST}$ of the CIoT network is defined as

(14) $$P_{SST}=P_{r}\left(R_{S}>0,P_{I1}^{Int-PS}\leq \Gamma ,P_{I2}^{Int-PS}\leq \Gamma \right),$$

where Γ is the interference threshold of *P*.

From the expressions of $R_{S}^{PS}$, $P_{I1}^{Int-PS}$, and $P_{I2}^{Int-PS}$, we can find out that the event $P_{I1}^{Int-PS}\leq \Gamma$ is independent from the events $R_{S}^{PS}>0$ and $P_{I2}^{Int-PS}\leq \Gamma$, respectively. Thus, the expression of $P_{SST}^{Int-PS}$ can be written as

(15) $$P_{SST}^{Int-PS}=P_{r}\left(R_{S}^{PS}>0,P_{I2}^{Int-PS}\leq \Gamma \right)P_{r}\left(P_{I1}^{Int-PS}\leq \Gamma \right).$$

In the following, we will calculate $P_{r}\left(P_{I1}^{Int-PS}\leq \Gamma \right)$ and $Pr(R_{S}^{PS}>0,P_{I2}^{Int-PS}<\Gamma )$ separately:

(16) $$\begin{array}{cc} P_{r}\left(P_{I1}^{Int-PS}\leq \Gamma \right) & =P_{r}(P_{S}|h_{SP}{|}^{2}+P_{D}|h_{DP}{|}^{2}+\sigma_{P1}^{2}\leq \Gamma )\\ & =P_{r}\left(X_{1}+X_{2}\leq u\right), \end{array}$$

where $X_{1}=P_{S}{\left|h_{SP}\right|}^{2}$, $X_{2}=P_{D}{\left|h_{DP}\right|}^{2}$, and $u=\Gamma -\sigma_{P1}^{2}$. Since $X_{1}$ and $X_{2}$ are exponentially distributed random variables with rate parameter $\lambda_{1}$ and $\lambda_{2}$, the probability density function of $X_{1}+X_{2}$ is calculated as

(17) $$f_{X_{1}+X_{2}}\left(x\right)=\left\{\begin{array}{cc} \lambda_{1}^{2}x\exp\left(-\lambda_{1}x\right) & \lambda_{1}=\lambda_{2},\\ \frac{\lambda_{1}\lambda_{2}(\exp\left(-\lambda_{1}x\right)-\lambda_{1}\exp\left(-\lambda_{2}x\right)}{\lambda_{2}-\lambda_{1}} & \lambda_{1}\neq \lambda_{2}, \end{array}\right.$$

where $\lambda_{1}=1/\left(P_{S}g_{SP}\right)$, $\lambda_{2}=1/\left(P_{D}g_{DP}\right)$. Using (17) in (16), we can get

(18) $$P_{r}\left(P_{I1}^{Int-PS}\leq \Gamma \right)=\int_{0}^{u}f_{X_{1}+X_{2}}\left(x\right)dx.$$

By evaluating the integral in (18), we can get

(19) $$P_{r}\left(P_{I1}^{Int-PS}\leq \Gamma \right)=\left\{\begin{array}{cc} 1-\left(1+\lambda_{1}u\right)\exp\left(-\lambda_{1}u\right)) & \lambda_{1}=\lambda_{2},\\ 1-\frac{\lambda_{2}\exp\left(-\lambda_{1}u\right)-\lambda_{1}\exp\left(-\lambda 2u\right)}{\lambda_{2}-\lambda_{1}} & \lambda_{1}\neq \lambda_{2}. \end{array}\right.$$

The second term of the right side of (15) can be calculated as follows:

(20) $$\begin{array}{c} P_{r}\left(R_{S}^{PS}>0,P_{I2}^{Int-PS}\leq \Gamma \right)\\ =P_{r}(\gamma_{D}^{Int-PS}>\gamma_{R}^{Int-PS},\eta \rho (P_{S}|h_{SR}{|}^{2}+P_{D}|h_{DR}{|}^{2})|h_{RP}{|}^{2}+\sigma_{P2}^{2}\leq \Gamma )\\ =\int_{0}^{+\infty }P_{r}\{\left[\eta \rho P_{D}t^{2}-\sigma_{D}^{2}\left(1-\rho \right)\right]P_{S}|h_{SR}{|}^{2}>u_{1},P_{S}|h_{SR}{|}^{2}+P_{D}t\leq \frac{v}{|h_{RP}{|}^{2}}\}\lambda_{3}\exp\left(-\lambda_{3}t\right)dt\\ =\int_{0}^{\sqrt{\frac{\sigma_{D}^{2}\left(1-\rho \right)}{\eta \rho P_{D}}}}\int_{0}^{w}\lambda_{3}\lambda_{5}\exp\left(-\lambda_{3}t-\lambda_{5}x\right)\left(1-\exp\left(-\frac{\lambda_{4}v}{x+P_{D}t}\right)\right)dxdt\\ +\int_{\sqrt{\frac{\sigma_{D}^{2}\left(1-\rho \right)}{\eta \rho P_{D}}}}^{+\infty }\int_{w}^{+\infty }\lambda_{3}\lambda_{5}\exp\left(-\lambda_{3}t-\lambda_{5}x\right)\left(1-\exp\left(-\frac{\lambda_{4}v}{(x+P_{D}t}\right)\right)dxdt, \end{array}$$

where $u_{1}=\sigma_{D}^{2}\left(1-\rho \right)P_{D}t-\eta \rho P_{D}^{2}t^{3}+\sigma_{R}^{2}\sigma_{D}^{2}$, $v=(\Gamma -\sigma_{P2}^{2})/\eta \rho$, $w=u_{1}/\left(\eta \rho P_{D}t^{2}-\sigma_{D}^{2}\left(1-\rho \right)\right)$, $\lambda_{3}=1/g_{DR}$, $\lambda_{4}=1/g_{RP}$, $\lambda_{5}=1/\left(P_{S}g_{SR}\right)$. It is challenging to obtain a closed-form solution for the double integral (20); however, the problem can be numerically solved through computer simulation. Combining (19) and (20), $P_{SST}$ of Int-PS can be obtained.

### 4.2. Precoded Secure Scheme Based on PS Policy

From the derivation of $P_{SST}^{Int-PS}$, we can see that, as the channel quality between the IoT nodes gets better, the chance of outage in the second phase increases, which would degrade $P_{SST}$. In order to eliminate this effect, a precoded secure scheme based on PS policy (Pre-PS) is proposed to eliminate the influence of the channels.

#### 4.2.1. Energy Harvesting and Information Processing of Pre-PS

In the precoded scheme, the EH relay broadcasts training signals so that both *S* and *D* estimate the channels between them and *R*. The transmit signals from *S* and *D* are

(21) $$x_{S}^{\prime }=h_{SR}^{-1}x_{S},\;x_{D}^{\prime }=h_{DR}^{-1}x_{D}.$$

For the precoded secure scheme, the transmit power at *R* becomes

(22) $$P_{H}=\eta \rho \left(P_{S}+P_{D}\right).$$

(1−ρ) of the received signal in the first phase is used for information processing at *R*, and it can be written as

(23) $$y_{R}=\sqrt{\left(1-\rho \right)}\left(\sqrt{P_{S}}x_{S}+\sqrt{P_{D}}x_{D}\right)+n_{R}.$$

Based on (23), the SINR at *R* can be written as

(24) $$\gamma_{R}^{Pre-PS}=\frac{\left(1-\rho \right)P_{S}}{\left(1-\rho \right)P_{D}+\sigma_{R}^{2}}.$$

Similarly, the received signal at *P* is written as

(25) $$y_{P1}=\sqrt{P_{S}}\frac{h_{SP}}{h_{SR}}x_{S}+\sqrt{P_{D}}\frac{h_{DP}}{h_{DR}}x_{D}+n_{P1},$$

and the instantaneous interference at *P* is given as

(26) $$P_{I1}^{Pre-PS}=P_{S}\frac{|h_{SP}{|}^{2}}{|h_{SR}{|}^{2}}+P_{D}\frac{|h_{DP}{|}^{2}}{|h_{DR}{|}^{2}}+\sigma_{P1}^{2}.$$

In the second phase, amplify-and-forward relaying protocol is used at *R*, and the transmit signal of *R* is expressed as $x_{R}=\beta y_{R}$, where $\beta =\sqrt{\frac{P_{H}}{\left(1-\rho \right)\left(P_{S}+P_{D}\right)+\sigma_{R}^{2}}}$. After performing interference self-cancellation, the received signal $y_{D}$ at *D* becomes

(27) $$y_{D}=\beta \sqrt{\left(1-\rho \right)P_{S}}h_{DR}x_{S}+\beta h_{DR}n_{R}+n_{D},$$

and the received SNR at *D* is

(28) $$\gamma_{D}^{Pre-PS}=\frac{\eta \rho \left(1-\rho \right)P_{S}{\left|h_{DR}\right|}^{2}}{\eta \rho |h_{DR}{|}^{2}\sigma_{R}^{2}+\left(1-\rho \right)\sigma_{D}^{2}+\frac{\sigma_{R}^{2}\sigma_{D}^{2}}{P_{S}+P_{D}}}.$$

Similarly, we can get the received power of interference plus AWGN at *P* as

(29) $$P_{I2}^{Pre-PS}=\eta \rho \left(P_{S}+P_{D}\right){\left|h_{RP}\right|}^{2}+\sigma_{P2}^{2}.$$

#### 4.2.2. Probability of Successfully Secure Transmission of Pre-PS

From the expressions of $R_{S}^{PS}$, $P_{I1}^{Pre-PS}$, and $P_{I2}^{Pre-PS}$, we can find out that the event $P_{I2}^{Pre-PS}\leq \Gamma$ is independent from the events $R_{S}^{PS}>0$ and $P_{I1}^{Pre-PS}\leq \Gamma$, respectively. Thus, the expression of $P_{SST}^{Pre-PS}$ becomes

(30) $$P_{SST}^{Pre-PS}=P_{r}\left(R_{S}>0,P_{I1}^{Pre-PS}\leq \Gamma \right)P_{r}\left(P_{I2}^{Pre-PS}\leq \Gamma \right).$$

In the following, we will formulate the $P_{SST}^{Pre-PS}$ in two steps. Firstly,

(31) $$\begin{array}{cc} P_{r}\left(P_{I2}^{Pre-PS}\leq \Gamma \right) & =P_{r}(\eta \rho \left(P_{S}+P_{D}\right)|h_{RP}{|}^{2}+\sigma_{P2}^{2}\leq \Gamma )\\ & =1-\exp(-\lambda_{4}u_{2}), \end{array}$$

where $u_{2}=\frac{\Gamma -\sigma_{P2}^{2}}{\eta \rho (P_{S}+P_{D})}$. Secondly,

(32) $$\begin{array}{cc} P_{r}\left(R_{S}^{PS}>0,P_{I1}^{Pre-PS}\leq \Gamma \right) & =P_{r}\left(\gamma_{D}^{Pre-PS}>\gamma_{R}^{Pre-PS},\frac{P_{S}{\left|h_{SP}\right|}^{2}}{|h_{SR}{|}^{2}}+\frac{P_{D}{\left|h_{DP}\right|}^{2}}{|h_{DR}{|}^{2}}+\sigma_{P1}^{2}\leq \Gamma \right)\\ & =P_{r}\left((\right|h_{DR}{|}^{2}>\theta ,\frac{P_{S}{\left|h_{SP}\right|}^{2}}{|h_{SR}{|}^{2}}+\frac{P_{D}{\left|h_{DP}\right|}^{2}}{|h_{DR}{|}^{2}}\leq u)||h_{DR}{|}^{2}=t)P_{r}\left(\right|h_{DR}{|}^{2}=t)\\ & =\int_{\theta }^{+\infty }P_{r}\left(\frac{P_{S}{\left|h_{SP}\right|}^{2}}{|h_{SR}{|}^{2}}+\frac{P_{D}{\left|h_{DP}\right|}^{2}}{t}\leq u\right)f_{|h_{DR}{|}^{2}}\left(t\right)dt, \end{array}$$

where $\theta =\frac{\left(\left(1-\rho \right)P_{S}+\left(1-\rho \right)P_{D}+\sigma_{R}^{2}\right)\sigma_{D}^{2}}{\eta \rho \left(1-\rho \right)\left(P_{S}+P_{D}\right)P_{D}}$. In addition,

(33) $$\begin{array}{c} P_{r}\left(\frac{P_{S}{\left|h_{SP}\right|}^{2}}{|h_{SR}{|}^{2}}+\frac{P_{D}{\left|h_{DP}\right|}^{2}}{t}\leq u\right)=P_{r}\left(X+Y\leq u\right)\\ =\int_{0}^{u}\int_{0}^{u}\frac{\lambda_{1}\lambda_{6}}{{\left(\lambda_{1}x+\lambda_{6}\right)}^{2}}\lambda_{2}t\exp\left(-\lambda_{2}ty\right)dxdy\\ =\int_{0}^{u}\lambda_{2}t\exp\left(-\lambda_{2}ty\right)\left(1+\frac{\lambda_{6}}{\lambda_{1}y-\lambda_{6}-\lambda_{1}u}\right)dy, \end{array}$$

where $\lambda_{6}=1/g_{SR}$. Substituting (33) in (32) and exchanging the order of integration, we get

(34) $$\begin{array}{c} P_{r}\left(R_{S}^{PS}>0,P_{I1}^{Pre-PS}\leq \Gamma \right)\\ =\underset{①}{\underset{︸}{\int_{0}^{u}\frac{\lambda_{3}\lambda_{2}\theta \exp\left(-\theta \left(\lambda_{2}y+\lambda_{3}\right)\right)}{\lambda_{2}y+\lambda_{3}}dy}}+\underset{②}{\underset{︸}{\int_{0}^{u}\frac{\lambda_{3}\lambda_{2}\exp\left(-\theta \left(\lambda_{2}y+\lambda_{3}\right)\right)}{{\left(\lambda_{2}y+\lambda_{3}\right)}^{2}}dy}}\\ +\underset{③}{\underset{︸}{\int_{0}^{u}\frac{\lambda_{6}\lambda_{3}\lambda_{2}\theta \exp\left(-\theta \left(\lambda_{2}y+\lambda_{3}\right)\right)}{\left(\lambda_{1}y-\lambda_{6}-\lambda_{1}u\right)\left(\lambda_{2}y+\lambda_{3}\right)}dy}}+\underset{④}{\underset{︸}{\int_{0}^{u}\frac{\lambda_{6}\lambda_{3}\lambda_{2}\exp\left(-\theta \left(\lambda_{2}y+\lambda_{3}\right)\right)}{\left(\lambda_{1}y-\lambda_{6}-\lambda_{1}u\right){\left(\lambda_{2}y+\lambda_{3}\right)}^{2}}dy}}. \end{array}$$

By making use of the method of partial fraction expansion, ③ and ④ can be reduced as

(35) $$\begin{array}{c} ③=\frac{\lambda_{3}\lambda_{6}\theta }{\lambda_{6}+\lambda_{1}u+\lambda_{1}\lambda_{3}/\lambda_{2}}\left\{\int_{0}^{u}\frac{\exp(-\theta \left(\lambda_{2}y+\lambda_{3}\right))}{y-(\lambda_{6}/\lambda_{1}+u)}dy-\int_{0}^{u}\frac{\exp(-\theta \left(\lambda_{2}y+\lambda_{3}\right))}{y+\lambda_{3}/\lambda_{2}}dy\right\}\\ ④=\frac{\lambda_{1}\lambda_{3}\lambda_{6}\lambda_{2}}{{\left(\lambda_{6}+\lambda_{1}u+\lambda_{1}\lambda_{3}/\lambda_{2}\right)}^{2}}\left\{\int_{0}^{u}\frac{\exp(-\theta \left(\lambda_{2}y+\lambda_{3}\right))}{y-(\lambda_{6}/\lambda_{1}+u)}dy-\int_{0}^{u}\frac{\exp(-\theta \left(\lambda_{2}y+\lambda_{3}\right))}{y+\lambda_{3}/\lambda_{2}}dy\right\}\\ -\frac{\lambda_{1}\lambda_{3}\lambda_{6}/\lambda_{2}}{\lambda_{6}+\lambda_{1}u+\lambda_{1}\lambda_{3}/\lambda_{2}}\int_{0}^{u}\frac{\exp(-\theta \left(\lambda_{2}y+\lambda_{3}\right))}{{\left(y+\lambda_{3}/\lambda_{2}\right)}^{2}}dy. \end{array}$$

Using the Equation (3.353.3) of [39], we have

(36) $$\begin{array}{c} \int_{0}^{u}\frac{\exp(-\theta \left(\lambda_{2}y+\lambda_{3}\right))}{\lambda_{2}y+\lambda_{3}}dy=\left[ei\left(-\lambda_{2}\theta u-\lambda_{3}\theta \right)-ei\left(-\lambda_{3}\theta \right)\right]/\lambda_{2}\\ \int_{0}^{u}\frac{\exp(-\theta \left(\lambda_{2}y+\lambda_{3}\right))}{{\left(\lambda_{2}y+\lambda_{3}\right)}^{2}}dy=\frac{\exp(-\lambda_{3})}{\lambda_{2}\lambda_{3}}-\frac{\exp(-\left(\lambda_{3}\theta +\lambda_{2}\theta u\right))}{\left(\lambda_{3}+\lambda_{2}u\right)\lambda_{2}^{2}}-\frac{\theta }{\lambda_{2}}\left[ei\left(-\lambda_{2}\theta u-\lambda_{3}\theta \right)-ei\left(-\lambda_{3}\theta \right)\right]\\ \int_{0}^{u}\frac{\exp(-\theta \left(\lambda_{2}y+\lambda_{3}\right))}{y-(\lambda_{6}/\lambda_{1}+u)}dy=\exp\left(-\theta \left(\lambda_{3}+\lambda_{6}\lambda_{2}/\lambda_{1}+\lambda_{2}u\right)\right)\left[ei\left(\lambda_{2}\lambda_{6}\theta /\lambda_{1}\right)-ei\left(\lambda_{2}\lambda_{6}\theta /\lambda_{1}+\lambda_{2}\theta u\right)\right], \end{array}$$

where ei(x) is the exponential integral, and $ei\left(x\right)=-\int_{-x}^{\infty }\left(\exp\left(-t\right)/t\right)dt$. By substituting (36) into (34) and combining similar terms, we can get

(37) $$\begin{array}{c} P_{r}\left(R_{S}>0,P_{I1}^{Pre-PS}\leq \Gamma \right)\\ =\frac{\lambda_{3}\lambda_{6}/\left(\lambda_{1}\lambda_{2}\right)}{{\left(\lambda_{3}/\lambda_{2}+\lambda_{6}/\lambda_{1}+u\right)}^{2}}E_{1}+\left(\frac{\lambda_{3}\lambda_{6}/\left(\lambda_{1}\lambda_{2}\right)}{{\left(\lambda_{3}/\lambda_{2}+\lambda_{6}/\lambda_{1}+u\right)}^{2}}+\frac{\lambda_{3}\lambda_{6}\theta /\lambda_{1}}{\lambda_{3}/\lambda_{2}+\lambda_{6}/\lambda_{1}+u}\right)\\ \exp\left(-\lambda_{3}\theta -\left(\frac{\lambda_{6}}{\lambda_{1}}+u\right)\lambda_{2}\theta \right)E_{2}+\exp\left(-\lambda_{3}\theta \right)\left(\frac{\lambda_{3}}{\lambda_{2}}-\frac{\lambda_{3}\lambda_{6}/\left(\lambda_{1}\lambda_{2}\right)}{\lambda_{3}/\lambda_{2}+\lambda_{6}/\lambda_{1}+u}\right)\left(\frac{\lambda_{2}}{\lambda_{3}}-\frac{\exp(-\lambda_{2}\theta u)}{\lambda_{3}/\lambda_{2}+u}\right), \end{array}$$

where $E_{1}=ei\left(-\lambda_{3}\theta \right)-ei\left(-\lambda_{2}\theta u-\lambda_{3}\theta \right)$ and $E_{2}=ei\left(\lambda_{6}\lambda_{2}\theta /\lambda_{1}\right)-ei\left(\left(\lambda_{6}/\lambda_{1}+u\right)\lambda_{2}\theta \right)$, respectively.

Therefore, the analytical expression of $P_{SST}^{Pre-PS}$ is obtained as follows:

(38) $$\begin{array}{cc} P_{SST}^{Pre-PS}= & \left[1-\exp\left(-\lambda_{4}u_{2}\right)\right][\frac{a/\lambda_{2}}{b^{2}}E_{1}+\left(\frac{a/\lambda_{2}}{b^{2}}+\frac{a\theta }{b}\right)\exp\left(-\lambda_{3}\theta -\left(\frac{\lambda_{6}}{\lambda_{1}}+u\right)\lambda_{2}\theta \right)E_{2}\\ & +\left(\frac{\lambda_{3}}{\lambda_{2}}-\frac{a/\lambda_{2}}{b}\right)\left(\frac{\lambda_{2}}{\lambda_{3}}-\frac{\exp(-\lambda_{2}\theta u)}{\lambda_{3}/\lambda_{2}+u}\right)\exp\left(-\lambda_{3}\theta \right)], \end{array}$$

where $a=\lambda_{3}\lambda_{6}/\lambda_{1}$, $b=\lambda_{3}/\lambda_{2}+\lambda_{6}/\lambda_{1}+u$.

## 5. Secure Schemes Based on TS Policy

**Figure 4:**
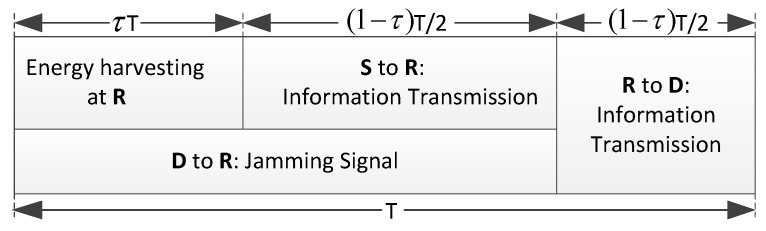
The TS policy.

The TS-policy-based relaying protocol is shown in Figure 4, where the first τT duration (0<τ<1) is used by *R* to harvest energy from the received signals, while the rest (1−τ)T duration is further split into two equal subslots, each of duration (1−τ)T/2. τ is the time splitting ratio. In the first subslot, *S* transmits the information, and *D* transmits a jamming signal simultaneously. In the second subslot, *R* amplifies and forwards the received signal to *D*. An intuitive secure scheme based on TS policy (Int-TS) is illustrated as follows.

### 5.1. Intuitive Secure Scheme Based on TS Policy

#### 5.1.1. Energy Harvesting and Information Processing of Int-TS

For the TS policy described above, the harvested energy $E_{H}$ at *R* is given as

(39) $$E_{H}=\eta \tau TP_{D}{\left|h_{DR}\right|}^{2}.$$

Then, this harvested energy is used by *R* to forward the information, and the transmit power is

(40) $$P_{H}=\frac{E_{H}}{\frac{(1-\tau )T}{2}}=\frac{2\eta \tau P_{D}{\left|h_{DR}\right|}^{2}}{1-\tau }.$$

In the first subslot, *S* and *D* transmit to *R* at the same time. Both the information signal and jamming signal are received by *R*, which can be expressed as

(41) $$y_{R}=\sqrt{P_{S}}h_{SR}x_{S}+\sqrt{P_{D}}h_{DR}x_{D}+n_{R}.$$

Based on (41), the received SINR at *R* can be written as

(42) $$\gamma_{R}^{Int-TS}=\frac{P_{S}{\left|h_{SR}\right|}^{2}}{P_{D}{\left|h_{DR}\right|}^{2}+\sigma_{R}^{2}}.$$

Similarly, the received signal $y_{P1}$ at *P* is written as

(43) $$y_{P1}=\sqrt{P_{S}}h_{SP}x_{S}+\sqrt{P_{D}}h_{DP}x_{D}+n_{P1},$$

and the instantaneous interference power to *P* is

(44) $$P_{I1}^{Int-TS}=P_{S}|h_{SP}{|}^{2}+P_{D}{\left|h_{DP}\right|}^{2}+\sigma_{P1}^{2}.$$

In the second subslot, *R* forwards the amplified version $x_{R}=\beta y_{R}$ to *D*, where $\beta =\sqrt{\frac{P_{H}}{P_{S}|h_{SR}{|}^{2}+P_{D}{\left|h_{DR}\right|}^{2}+\sigma_{R}^{2}}}$. By subtracting the self-interference term, the resultant received signal $y_{D}$ at *D* becomes

(45) $$y_{D}=\beta \sqrt{P_{S}}h_{DR}h_{SR}x_{S}+\beta h_{DR}n_{R}+n_{D}.$$

The SNR at *D* can be written as

(46) $$\gamma_{D}^{Int-TS}=\frac{P_{S}|h_{DR}{|}^{2}{\left|h_{SR}\right|}^{2}}{|h_{DR}{|}^{2}\sigma_{R}^{2}+\frac{\left(1-\tau \right)(P_{S}|h_{SR}{|}^{2}+P_{D}|h_{DR}{|}^{2}+\sigma_{R}^{2})\sigma_{D}^{2}}{2\eta \tau P_{D}{\left|h_{DR}\right|}^{2}}}.$$

Finally, the instantaneous interference power to *P* in the second subslot can be expressed as

(47) $$P_{I2}^{Int-TS}=\frac{2\eta \tau P_{D}{\left|h_{DR}\right|}^{2}}{1-\tau }{\left|h_{RP}\right|}^{2}+\sigma_{P2}^{2}.$$

#### 5.1.2. Probability of Successfully Secure Transmission of Int-TS

The instantaneous rate of the TS policy is calculated by

(48) $$R_{S}^{TS}=\frac{1-\tau }{2}{\left[\log_{2}\left(\frac{1+\gamma_{D}}{(1+\gamma_{R}}\right)\right]}^{+},$$

where the coefficient (1−τ)/2 denotes the effective time of information transmission. Referring to the intuitive scheme based on PS policy, $P_{SST}^{Int-TS}$ can be expressed as

(49) $$P_{SST}^{Int-TS}=P_{r}\left(R_{S}^{TS}>0,P_{I2}^{Int-TS}\leq \Gamma \right)P_{r}\left(P_{I1}^{Int-TS}\leq \Gamma \right).$$

Note that the probability of the event $P_{I1}^{Int-TS}\leq \Gamma$ has been acquired in (19), and

(50) $$\begin{array}{cc} P_{r}\left(R_{S}^{TS}>0,P_{I2}^{Int-TS}\leq \Gamma \right) & =P_{r}\left(\gamma_{D}^{Int-TS}>\gamma_{R}^{Int-TS},\frac{2\eta \tau P_{D}|h_{DR}{|}^{2}{\left|h_{RP}\right|}^{2}}{1-\tau }+\sigma_{P2}^{2}\leq \Gamma \right)\\ & =P_{r}\left((\right|h_{SR}{|}^{2}<v_{1},|h_{RP}{|}^{2}\leq v_{2})||h_{DR}{|}^{2}=t)P_{r}\left(\right|h_{DR}{|}^{2}=t)\\ & =\int_{0}^{+\infty }(1-exp\left(-\lambda_{3}v_{1}\right)(1-exp\left(\lambda_{5}v_{2}\right)\lambda_{1}exp\left(-\lambda_{1}t\right)dt, \end{array}$$

where $v_{1}=2\eta \rho P_{D}^{2}t^{3}/\left(\left(1-\tau \right)P_{S}\sigma_{D}^{2}\right)-P_{D}^{2}t/P_{S}-\sigma_{R}^{2}/P_{S}$, and $v_{2}=\left(1-\tau \right)\left(\Gamma -\sigma_{P2}^{2}\right)/\left(2\eta \tau P_{D}t\right)$, respectively. As in Section 4.1.2, it is difficult to solve the above integral due to the complexity of the integrand function.

### 5.2. Precoded Secure Scheme Based on TS Policy

Owing to the AF protocol, the intuitive secure scheme based on TS policy encounters the same problem as the intuitive secure scheme based on PS policy. That is, the chance of outage in the second subslot increases as the channel quality of the link between *R* and *S* (*D*) improves. Therefore, taking into consideration of the channel influence, we propose the precoded scheme based on TS policy (Pre-TS) in the following.

#### 5.2.1. Energy Harvesting and Information Processing of Pre-TS

For Pre-TS, the transmit power at *R* is given as

(51) $$P_{H}=\frac{2\eta \tau P_{D}}{1-\tau },$$

while the received signal at *R* is given by

(52) $$\begin{array}{c} y_{R}=\sqrt{P_{S}}x_{S}+\sqrt{P_{D}}x_{D}+n_{R}. \end{array}$$

Based on (52), the SINR at *R* can be written as

(53) $$\gamma_{R}^{Pre-TS}=\frac{P_{S}}{P_{D}+\sigma_{R}^{2}}.$$

Similarly, we denote the received signal at *P* as

(54) $$\begin{array}{c} y_{P1}=\frac{\sqrt{P_{S}}h_{SP}}{h_{SR}}x_{S}+\frac{\sqrt{P_{D}}h_{DP}}{h_{DR}}x_{D}+n_{P1}. \end{array}$$

In addition, the instantaneous interference power plus the noise at *P* is given as

(55) $$P_{I1}^{Pre-TS}=\frac{P_{S}{\left|h_{SP}\right|}^{2}}{|h_{SR}{|}^{2}}+\frac{P_{D}{\left|h_{DP}\right|}^{2}}{|h_{DR}{|}^{2}}+\sigma_{P1}^{2}.$$

In the second subslot, *R* forwards the amplified version $x_{R}=\beta y_{R}$ to *D*, where $\beta =\sqrt{\frac{P_{H}}{P_{S}+P_{D}+\sigma_{R}^{2}}}.$ Then, the received signal at *D* is written as

(56) $$y_{D}=\beta \sqrt{P_{S}}h_{DR}x_{S}+\beta h_{DR}n_{R}+n_{D}.$$

Thus, by a series of simplifications, the SNR at *D* eventually becomes

(57) $$\gamma_{D}^{Pre-TS}=\frac{P_{S}{\left|h_{DR}\right|}^{2}}{|h_{DR}{|}^{2}\sigma_{R}^{2}+\frac{\left(1-\tau \right)\left(P_{S}+P_{D}+\sigma_{R}^{2}\right)\sigma_{D}^{2}}{2\eta \tau P_{D}}}.$$

Similarly, the instantaneous interference power plus the noise at *P* in the second subslot can be expressed as

(58) $$P_{I2}^{Pre-TS}=\frac{2\eta \tau P_{D}}{1-\tau }{\left|h_{RP}\right|}^{2}+\sigma_{P2}^{2}.$$

#### 5.2.2. Probability of Successfully Secure Transmission of Pre-TS

Imitating the formulation of $P_{SST}$ for Pre-PS, the expression of $P_{SST}^{Pre-TS}$ can be written as

(59) $$\begin{array}{cc} P_{SST} & =P_{r}\left(R_{S}^{TS}>0,P_{I1}^{Pre-TS}\leq \Gamma \right)P_{r}\left(P_{I2}^{Pre-TS}\leq \Gamma \right)\\ & =\left[1-\exp\left(-\lambda_{4}v_{3}\right)\right][\frac{a/\lambda_{2}}{b^{2}}E_{1}+\left(\frac{a/\lambda_{2}}{b^{2}}+\frac{a\theta^{\prime }}{b}\right)\exp\left(-\lambda_{3}\theta^{\prime }-\left(\frac{\lambda_{6}}{\lambda_{1}}+u\right)\lambda_{2}\theta^{\prime }\right)E_{2}\\ & +\left(\frac{\lambda_{3}}{\lambda_{2}}-\frac{a/\lambda_{2}}{b}\right)\left(\frac{\lambda_{2}}{\lambda_{3}}-\frac{\exp(-\lambda_{2}\theta^{\prime }u)}{\lambda_{3}/\lambda_{2}+u}\right)\exp\left(-\lambda_{3}\theta \right)], \end{array}$$

where $v_{3}=\frac{\left(1-\tau \right)\left(\Gamma -\sigma_{P2}^{2}\right)}{2\eta \tau P_{D}}$, and $\theta^{\prime }=\left(1-\tau \right)\left(P_{S}+P_{D}+\sigma_{R}^{2}\right)\sigma_{D}^{2}/\left(2\eta \tau P_{D}^{2}\right)$. The detailed derivation follows the same steps as in Section 4.2.2. Thus, we skip the process here for brevity.

## 6. Discussion and Simulations

Numerical results are presented in this section to investigate the secrecy performance of the CIoT network where an untrusted wireless EH relay is used to help the secondary transmission. We discuss the influence of different system parameters on the probabilities of successfully secure transmission of the intuitive and precoded secure schemes based on PS and TS policies. Unless otherwise explicitly specified, simulation parameters are set as η=0.5, $\sigma_{D}^{2}=\sigma_{R}^{2}=\sigma_{P1}^{2}=\sigma_{P2}^{2}=\sigma^{2}$, $\Gamma /\sigma^{2}=23$ dB, $g_{SP}=g_{DP}=g_{RP}$ and $g_{SR}=g_{DR}$. Let $\alpha =g_{SR}/g_{SP}$. We define the transmit SNR of *S* and *D* for intuitive secure schemes as $P_{S}/\sigma^{2}=20dB$ and $P_{D}/\sigma^{2}=20$ dB, respectively.

### 6.1. Comparison between Int-PS and Pre-PS

In this subsection, the influence of power splitting factor ρ on $P_{SST}$ is discussed for the intuitive and precoded secure schemes based on PS policy. The channels are assumed independently identically distributed, and $g_{c}=1$. For a certain transmission, the transmit SNR for the precoded secure schemes is denoted as $P_{i}/\sigma^{2}{\left|h_{iR}\right|}^{2}$ (i∈{S,D}). The average transmit SNR is referred to the ratio of the average transmit power to the noise power for the duration of *T*, which represents the average power consumption.

**Figure 5:**
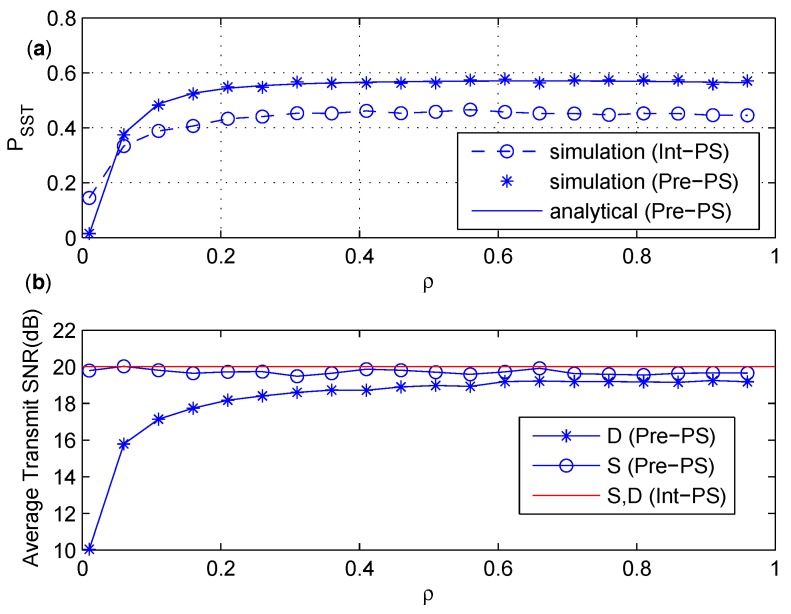
(**a**) $P_{SST}$ under various ρ. (**b**) The average transmit SNR under various ρ.

In Figure 5, the influence of ρ on $P_{SST}$ of the precoded and intuitive schemes based on PS policy is given, and the average power consumption is also discussed. First, we can find out from Figure 5a that the closed form expression of $P_{SST}$ of Pre-PS coincides with the simulation result perfectly. For the transmissions of Pre-PS, $P_{i}/\sigma_{i}^{2}{\left|h_{iR}\right|}^{2}$ (i∈{S,D}) is set to be 17 dB. We can see from Figure 5b that the average transmit SNR of Pre-PS at *D* during *T* is almost the same as that of Int-PS, which is 20 dB. The average transmit SNR of Pre-PS at *S* increases as ρ increases, and approaches 20 dB when ρ>0.6. From Figure 5, we find out that Pre-PS outperforms Int-PS in terms of $P_{SST}$ when the average power consumptions of both are similar. In addition, it is necessary to state that $P_{SST}$ of Pre-PS decreases dramatically and becomes worse than Int-PS when ρ approaches zero. This is mainly because the probability of the event $P_{I2}\leq \Gamma$ decreases dramatically and converges to zero when the value of ρ goes to zero, which can be found easily from (31).

**Figure 6:**
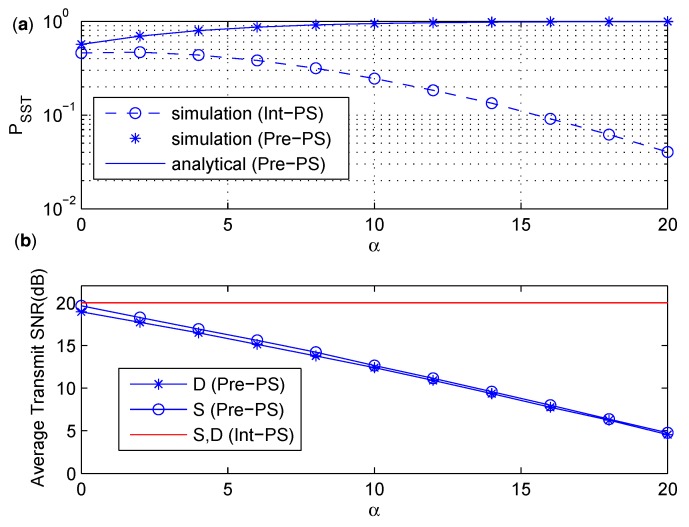
(**a**) $P_{SST}$ under various α. (**b**) The average transmit SNR under various α.

In Figure 6, the influence of different channel qualities on $P_{SST}$ is shown and the average power consumption is discussed. In the simulation , $g_{SR}$ and $g_{RD}$ increases so that α increases. We can see from Figure 6a that the analytical result of Pre-Ps is in excellent agreement with the simulation result of Pre-PS. The value of $P_{SST}$ of Pre-PS increases and converges to 1 quickly as α increases, while the situation is opposite for Int-PS. The reason is as follows. The increasing of α means better channel condition for the S−R and R−D links. As the transmit SNR for Int-PS is fixed, better channel quantity of S−R and R−D links would result in higher probability of interrupting the primary receiver in the second phase, which makes $P_{SST}$ get worse for Int-PS. In Pre-PS, the transmit power from *S* and *D* is adjusted according to the channel quality of S−R and R−D links, respectively. Therefore, the average transmit power from *S* and *D* of Pre-PS decreases as α increases as shown in Figure 6b. When α becomes larger, it is less possible to interfere the primary receiver in the first phase, and $P_{SST}$ of Pre-PS increases accordingly.

### 6.2. Discussion on the Effect of Power Splitting Ratio

**Figure 7:**
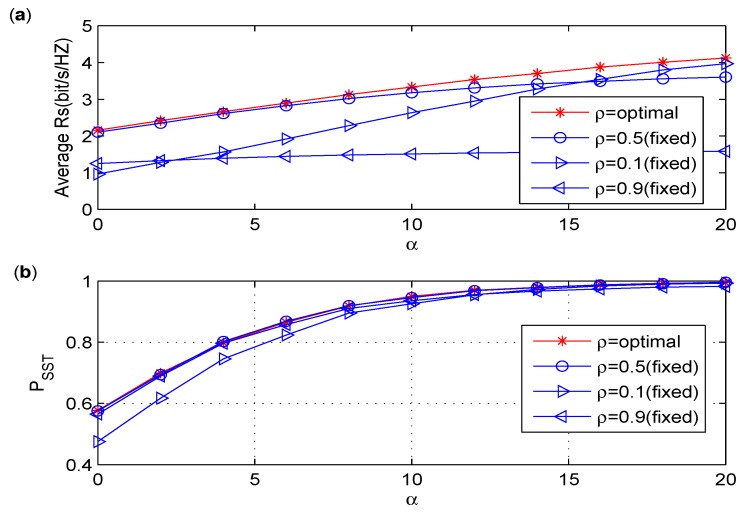
(**a**) Average Rs versus α for various ρ. (**b**) $P_{SST}$ versus α for various ρ.

From the above simulation results, we find out that Pre-PS outperforms Int-PS in terms of $P_{SST}$ under the similar average power consumption, and $P_{SST}$ is not sensitive to the changes of ρ. Since $P_{SST}$ only reflects the probability that a positive secrecy rate (Rs) is achieved under the primary interference constraint, although $P_{SST}$ almost stays the same when ρ>0.2, it is interesting to see whether there is a certain ρ to maximize the achievable secrecy rate while maintaining the maximum $P_{SST}$. Therefore, in Figure 7, we study the effect of ρ on the achievable secrecy rate of Pre-PS in the CIoT network.

As depicted in Figure 7, it is clear that the average Rs for ρ=0.5 is better than that for ρ=0.9, while the value of $P_{SST}$ keeps the same for ρ=0.5 and ρ=0.9. In the simulations, the optimal ρ is obtained by computer searching. Specifically, for each α, searching the optimal value of ρ to maximize Rs while satisfying the interference constraints. The average Rs for the optimal ρ is the best among those for different values of ρ. We should notice that the average Rs for ρ=0.5 is almost the same as that for the optimal ρ when α<10, and it is slightly worse than the optimal Rs when α becomes even larger. Therefore, in the following simulations regarding Pre-PS, ρ is set to 0.5.

### 6.3. Comparison between Int-TS and Pre-TS

In this subsection, we will compare Int-TS and Pre-TS in terms of $P_{SST}$, and discuss the influence of the time splitting factor τ and the channel gain factor α on $P_{SST}$. The transmit SNRs at *S* and *D* are denoted as $\frac{\left(1-\tau \right)P_{S}}{2\sigma^{2}}$, $\frac{\left(1+\tau \right)P_{D}}{2\sigma^{2}}$ for Int-PS and $\frac{\left(1-\tau \right)P_{S}}{2|h_{SR}{|}^{2}\sigma^{2}}$, $\frac{\left(1+\tau \right)P_{D}}{2|h_{DR}{|}^{2}\sigma^{2}}$ for Pre-PS, respectively.

**Figure 8:**
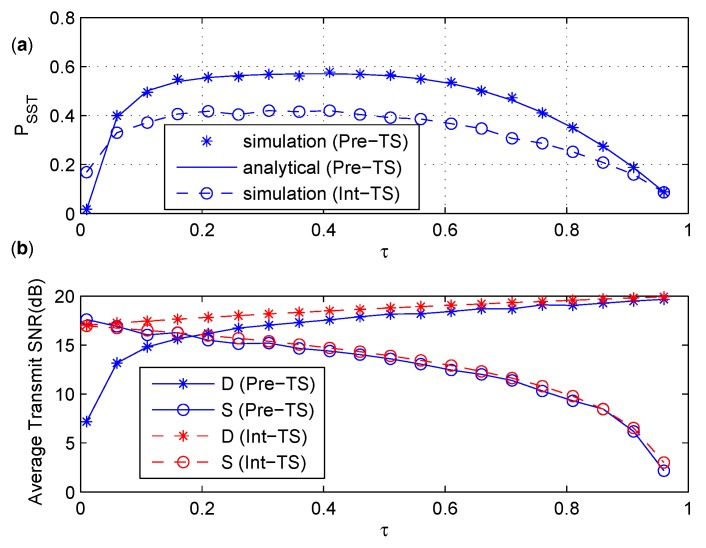
(**a**) $P_{SST}$ under various τ. (**b**) The average transmit SNR under various τ.

In Figure 8a, we compare $P_{SST}$ versus τ for both Int-TS and Pre-TS, in which simulation and analytical results about $P_{SST}$ are coincident for Pre-TS. Figure 8b shows the average power consumption during *T* at S and D for both Int-TS and Pre-TS. From Figure 8, one can see that Pre-TS outperform Int-TS in terms of $P_{SST}$ when the average power consumption of both is similar. When ρ approaches zero, $P_{SST}$ of Pre-TS decreases dramatically and becomes worse than that of Int-TS. This is mainly because the probability of the event $P_{I2}\leq \Gamma$ converges to zero when the value of ρ converges to zero, which can be seen easily from (59). From Figure 8a, we can see that there is an initial increase in $P_{SST}$ of Int-TS and Pre-TS as τ increases from zero, and then a fall in $P_{SST}$ when τ further increases from 0.4. The reason is that the relay harvests more energy as τ increases, and this in turn increases the relay’s transmit power. In this way, the information reception at the destination is improved and the received signal strength at the relay is degraded. However, once τ crosses a certain value, higher transmit power from the relay may break the primary interference constraint, and the poor signal strength at the relay delivers a negative effect on the secrecy rate. In the following simulations, τ=0.3 is used for Int-TS and Pre-TS.

**Figure 9:**
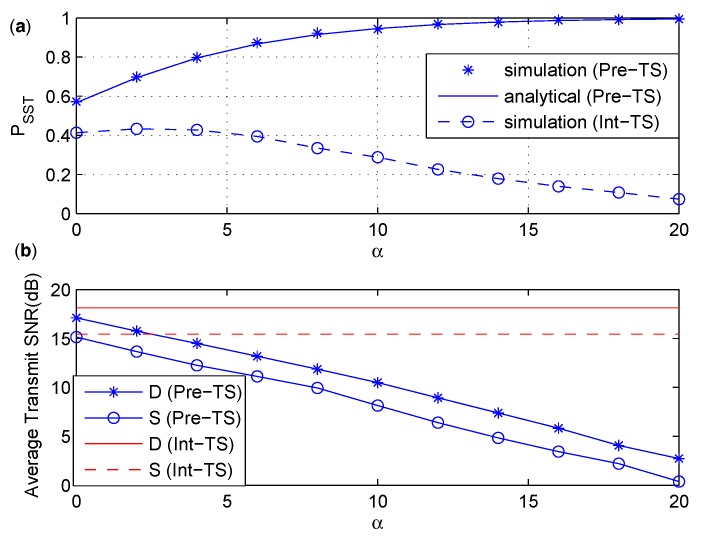
(**a**) $P_{SST}$ under various α. (**b**) The average transmit SNR under various α.

The influence of α on $P_{SST}$ is discussed in Figure 9a, and the average transmit power consumptions by *S* and *D* are given in Figure 9b. A similar situation as in Figure 6 can be found in Figure 9, where $P_{SST}$ increases as α increases for Pre-TS and decreases as α increases for Int-TS. This phenomenon can be explained in a similar way as in Figure 6. Since the transmit SNR for Int-TS is fixed, better channel quantity of S−R and R−D links would result in a higher possibility of interrupting the primary receiver in the second phase, which makes $P_{SST}$ get worse for Int-TS. However, the transmit power from *S* and *D* of Pre-TS is adjusted according to the channel quality of S−R and R−D links, respectively, so that the average transmit power of Pre-TS decreases as α increases, as shown in Figure 9b. Therefore, it is less possible to interfere the primary receiver in the first phase when α becomes larger, and $P_{SST}$ of Pre-TS increases as α increases. From Figure 9b, we can see that *D* always consumes more power than *S* for both Int-TS and Pre-TS, since it has to transmit power for energy harvesting in the period of τT.

### 6.4. Comparison between Pre-PS and Pre-TS

From the above discussion, we can see that the precoded secure scheme is better than the intuitive secure scheme based on both PS and TS policies. In this subsection, we compare the precoded schemes based on PS policy and TS policy, and mainly focus on the effects of the splitting factor ρ, τ, and interference temperature threshold Γ.

**Figure 10:**
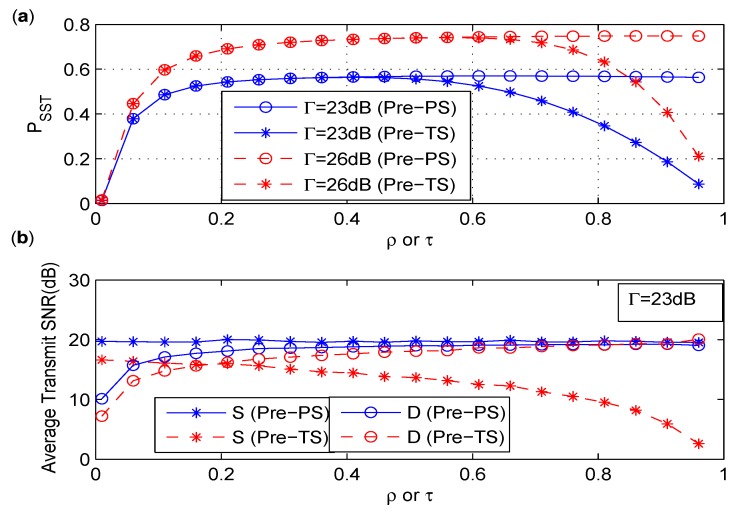
(**a**) $P_{SST}$ versus ρ or τ under different Γ. (**b**) The average transmit SNR versus ρ or τ under Γ=23dB.

As shown in Figure 10a, $P_{SST}$ initially increases and converges to a certain value eventually with the increase of ρ for Pre-PS. While for TS policy, $P_{SST}$ decreases gradually when τ is greater than a specified value. However, the maximum achievable values of $P_{SST}$ for Pre-PS and Pre-TS are the same. In addition, one can see that larger interference tolerance at the primary receiver results in better performance of $P_{SST}$. It can be found in Figure 10b that less power consumption happens at *S* for Pre-TS as the splitting factor increases. When Pre-PS and Pre-TS achieve the same maximum value of $P_{SST}$, the power consumption of *S* of Pre-TS is obviously lower than that of Pre-PS. Therefore, Pre-TS is more desirable than Pre-PS considering the energy constraint of the IoT device.

## 7. Conclusions

We have investigated the secrecy performance of a CIoT network where the secondary system utilizes a wireless EH untrusted node to help the transmission of the IoT device. Since the secondary destination can be equipped with adequate power supply, different secure schemes based on destination-aided jamming have been designed. The secrecy performance of the proposed secure schemes are evaluated in terms of $P_{SST}$, which is defined as the probability that the interference constraint of the primary user is satisfied and the secrecy rate is positive. The closed form of $P_{SST}$ for the precoded secure schemes based on PS and TS policies have been derived. The simulation results are coincident with the derived closed-form expressions perfectly, which validate the theoretical analysis presented in this paper. The numerical results reveal that, under similar transmit power consumption, the precoded secure schemes outperform the intuitive secure schemes in terms of $P_{SST}$. The precoded secure scheme based on TS policy is more energy efficient than that based on PS policy. Some useful design insights can be found from the numerical study of $P_{SST}$ under different system parameters. For example, $P_{SST}$ based on PS policy is not sensitive to the PS ratio ρ when ρ>0.2, and an optimal ρ maximizing the achievable secrecy rate can be found under the $P_{SST}$ constraint. The time splitting ratio τ in the TS policy shows both beneficial and harmful influences on the secure performance, and the optimal τ in the TS policy that maximizes $P_{SST}$ can be found.

As we know, an IoT network consists of a great number of IoT devices. In this paper, the secrecy performance is investigated for a fundamental scenario where a single secondary communication link is considered. It is of interest and importance to investigate practical scenarios where numerous IoT devices are expected. After we have evaluated the secrecy performance of the fundamental scenario, we can further study more practical scenarios that could involve power allocation and user scheduling designs. In our future work, we plan to study the secure communication of multiple IoT devices and secure multi-hop communication in an IoT network.

## Abbreviations

The following abbreviations are used in this manuscript:

IOT	The Internet of Things
CIOT	Cognitive IOT
EH	Energy harvesting
PS	Power splitting
TS	Time splitting
Int-PS/TS	The intuitive secure scheme based on PS/TS
Pre-PS/TS	The precoded secure scheme based on PS/TS
$P_{SST}$	The probability of successfully secure transmission
RATs	Radio access technologies
PLS	Physical layer security
CAE	Channel-aware encryption
CSRNs	Cognitive sensor radio networks
QoS	Quality-of-service
FD	Full-duplex
SPSC	Strictly positive secrecy capacity
AF	Amplify-and-forward
MF	Modulo-and-forward
